# A multimethod approach for county-scale geospatial analysis of emerging infectious diseases: a cross-sectional case study of COVID-19 incidence in Germany

**DOI:** 10.1186/s12942-020-00225-1

**Published:** 2020-08-13

**Authors:** Christopher Scarpone, Sebastian T. Brinkmann, Tim Große, Daniel Sonnenwald, Martin Fuchs, Blake Byron Walker

**Affiliations:** 1 Urban Forest Research and Ecological Disturbance (UFRED) Lab: Department of Geography, Ryerson University, 350 Victoria Street, Toronto, M5B 2K3 Canada; 2 Community Health Environments and Social Terrains (CHEST) Lab, Institut für Geographie, Friedrich-Alexander-Universität Erlangen-Nürnberg, Wetterkreuz 15, 91052 Erlangen, Germany

**Keywords:** COVID-19, SARS-CoV-2, GIS, Built environment, Socioeconomic status, Machine learning, Infectious disease, Exploratory Spatial Data Analysis (ESDA)

## Abstract

**Background:**

As of 13 July 2020, 12.9 million COVID-19 cases have been reported worldwide. Prior studies have demonstrated that local socioeconomic and built environment characteristics may significantly contribute to viral transmission and incidence rates, thereby accounting for some of the spatial variation observed. Due to uncertainties, non-linearities, and multiple interaction effects observed in the associations between COVID-19 incidence and socioeconomic, infrastructural, and built environment characteristics, we present a structured multimethod approach for analysing cross-sectional incidence data within in an Exploratory Spatial Data Analysis (ESDA) framework at the NUTS3 (county) scale.

**Methods:**

By sequentially conducting a geospatial analysis, an heuristic geographical interpretation, a Bayesian machine learning analysis, and parameterising a Generalised Additive Model (GAM), we assessed associations between incidence rates and 368 independent variables describing geographical patterns, socioeconomic risk factors, infrastructure, and features of the build environment. A spatial trend analysis and Local Indicators of Spatial Autocorrelation were used to characterise the geography of age-adjusted COVID-19 incidence rates across Germany, followed by iterative modelling using Bayesian Additive Regression Trees (BART) to identify and measure candidate explanatory variables. Partial dependence plots were derived to quantify and contextualise BART model results, followed by the parameterisation of a GAM to assess correlations.

**Results:**

A strong south-to-north gradient of COVID-19 incidence was identified, facilitating an empirical classification of the study area into two epidemic subregions. All preliminary and final models indicated that location, densities of the built environment, and socioeconomic variables were important predictors of incidence rates in Germany. The top ten predictor variables’ partial dependence exhibited multiple non-linearities in the relationships between key predictor variables and COVID-19 incidence rates. The BART, partial dependence, and GAM results indicate that the strongest predictors of COVID-19 incidence at the county scale were related to community interconnectedness, geographical location, transportation infrastructure, and labour market structure.

**Conclusions:**

The multimethod ESDA approach provided unique insights into spatial and aspatial non-stationarities of COVID-19 incidence in Germany. BART and GAM modelling indicated that geographical configuration, built environment densities, socioeconomic characteristics, and infrastructure all exhibit associations with COVID-19 incidence in Germany when assessed at the county scale. The results suggest that measures to implement social distancing and reduce unnecessary travel may be important methods for reducing contagion, and the authors call for further research to investigate the observed associations to inform prevention and control policy.

## Background

### COVID-19

Since the initial outbreak in late 2019 in Wuhan, China [1], the novel coronavirus SARS-CoV-2 has spread to 207 countries worldwide, causing an estimated 12.9 million cases and 569,128 deaths due to coronavirus disease 2019 (COVID-19), as of 13 of July [2]. In Germany, the first case was recorded on 27 of January 2020 [3], in Bavaria. Most recently there were 198,963 reported cases and 9064 deaths in Germany as of 13 of July 2020 [4]. Federal social distancing guidelines were nearing peak security measures on 28 March 2020, where curfews were being implemented independently at the NUTS-3 (county) level as early as 20 March [5].

Local person-to-person transmission of the virus is attributable to shedding on the nasopharyngeal, turbinate, and oropharyngeal surfaces [6, 7], then transmitted primarily via airborne droplets ejected from the nose or mouth [6]. Owing to an estimated average incubation period of 5-6 days and ranging up to two weeks [8–11], the virus can be transmitted to multiple persons by asymptomatic individuals [7]. Up to 78% of individuals who test positive are asymptomatic at the time of testing (Day, 2020), therefore likely accounting for the majority of new cases [7]. Research and public health guidelines have accordingly emphasised interpersonal proximity as a key risk factor, advising a minimum interpersonal distance of 1.5 m to reduce risk of transmission [11].

### Meta-population framework

**Fig. 1:**
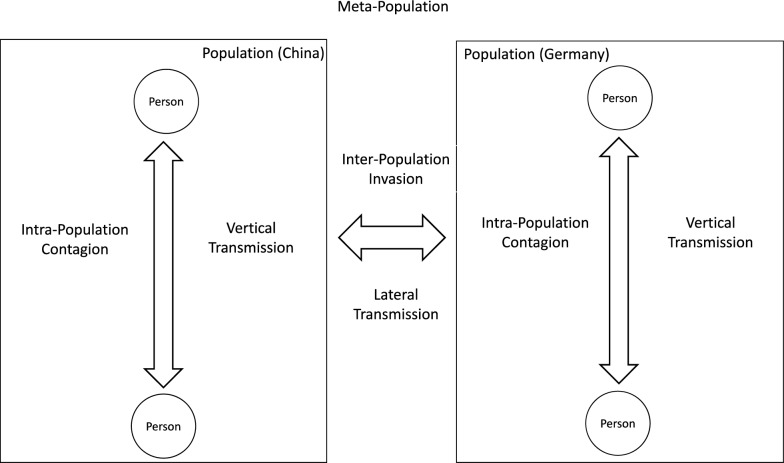
The meta-population framework describes the global and local transmission of emerging infectious diseases (EIDs) by inter-population invasion and intra-population contagion [15]

In order to identify spatial patterns and accurately model viral contagion a minimum number of infected individuals must be established. This threshold allows for the identification of transmission parameters necessary for deterministic modelling [12, 13]. Once patterns can be detected, the meta-population theory for epidemiology [14] provides a valuable framework for modelling and analysis. A meta-population is the aggregate of all global populations (Fig. 1). In the context of global CoV-SARS-2 spread, each country can be considered an individual population [12]. The transmission of CoV-SARS-2 is therefore broadly characterised by inter-population transmission and intra-population contagion.

Intra-population contagion [15, 16] can be locally driven, where individual members inside the extent of the initial outbreak boundary (Wuhan, China) begin to transmit the disease to other members of the local population. Should a threshold number of individuals be diagnosed, the local socioeconomic, built environment, and spatial patterns can then be analysed [13, 15]. The examination of these types of patterns and associations assists researchers and public health officials to define the spatial diffusion and reproduction of a disease, and accordingly, target prevention measures and direct interventions [17, 18].

The subsequent horizontal transmission is referred to as inter-population invasion [15, 19] and is characterised by a semi-stochastic process that acts on a global scale [15]. The infected members of the population transmit the virus from the outbreak extent to new uninfected cities between nodes of transportation networks such as airports and train stations [20, 21]. Global transmission of emerging infectious diseases (EIDs) is therefore the iterative process of intra-population contagion in a population that then allows a stochastic jump to inter-population invasion. We hypothesise that socioeconomic characteristics of a population and features of the built environment comprise important factors in both intra-population contagion and inter-population invasion (e.g., employment rates, social assistance, airports, and major train stations). By examining geospatial patterns of incidence and associated social- and built-environmental features across Germany, this cross-sectional study frames Germany as a population and each constituent county (NUTS-3) as an individual member of the population.

### Socioeconomic and built environment factors

Socioeconomic status (SES) is well understood to play a significant role in the transmission of infectious disease, for example, through intra-population contagion among socioeconomically homogeneous subpopulations [22]. For example, age plays a role both in individual risk of respiratory infection and in the frequency and nature of interpersonal contact [23]. More broadly, higher rates of infectious diseases such as influenza, invasive group A streptococcal infections, and pneumococcal infections have been observed among socioeconomically deprived subpopulations (e.g., low-income, high unemployment) [22]. Spatial analysis of SES has thus been widely used to investigate social and economic risk factors, predict high-risk areas, and target interventions [24, 25].

It is well understood that the built environment exerts an influence on patterns of human mobility and social interaction, which are in turn key factors in the transmission and prevalence of infectious disease [26]. For example, the aforementioned study on the risk respiratory infections indicates that the location of contact is important for the risk of transmission [23]. Furthermore, the spatial configuration of buildings can have an impact on disease transmission, for example, by affecting the density of persons moving through a confined space [26]. However, the density of features of the built environment has, to our knowledge, not yet been comprehensively modelled for spatial-epidemiological analysis of infectious disease, presenting an important avenue for investigation which this study seeks to begin to address.

### Geospatial analysis

Spatial epidemiology emphasises the importance of geographical patterns in understanding disease risk factors, incidence, and outcomes [17, 18]. For example, incidence rates of an infectious disease often exhibit spatial associations with SES and the built environment [18], which function as possible determinants of interpersonal contact and vulnerability to infection. The identification and investigation of geospatial patterns and high-/low-rate clusters is therefore a key process for characterising aetiologies, identifying high-risk populations, and targeting interventions [27].

The use of geographic information systems (GIS) facilitates empirical representation of the spatial associations between socioeconomic- and built environments and infectious disease incidence [17, 28]. Many studies focus on spatial autocorrelation, which provides a means of estimating the influence of proximity on the interactions between nearby features [28], both in that proximal features are more likely to interact and are more likely to be similar in composition [17, 29]. GIS thus provide a platform for modelling and analysing spatial autocorrelation within a spatial epidemiology framework [18], for example, by interpolating and examining spatiotemporal patterns of infectious disease [30] and identified associations with socioeconomic characteristics of subpopulations and relevant prevention and control measures [31].

Conversely, strictly mathematical approaches to epidemiological modelling focus predominantly on the simulation of propagation dynamics under various defined conditions [12, 15, 32]. These models focus on identifying transmission vectors and simulating transmission scenarios [32], and may include a spatial component [33]. As computational processing power continues to rapidly improve, researchers are increasingly able to incorporate sophisticated mathematical techniques, such as Bayesian machine learning, to model both geospatial patterns and socioeconomic/environmental data within a spatial-epidemiological framework [18, 34]. These efforts are key to identifying otherwise concealed geographical patterns and associations, an important initial step towards advancing our understanding of risk factors and transmission dynamics [27].

A rapid increase in the quantity of socioeconomic, environmental, and health data is further driving modern statistical methodologies for epidemiology modelling [35], as a growing number of variables must be modelled in order to more comprehensively explain spatial patterns of disease. Consequently, such methods are able to account for more complexity and thus have immense value for developing more informed decisions in health care and disease control [34]. Of particular prominence in recent years is the use of geospatially-explicit artificial intelligence for environmental epidemiology [36], including the combined use of machine learning, GIS, precision incidence data, and exposure modelling.

This cross-sectional study presents an empirical exploration and interpretation of the spatial patterns exhibited by COVID-19 incidence rates across Germany. A combination of epidemiological and machine learning techniques are used to identify associations between COVID-19 incidence rates and socioeconomic and built-environment characteristics at the county scale.

## Methods

**Fig. 2:**
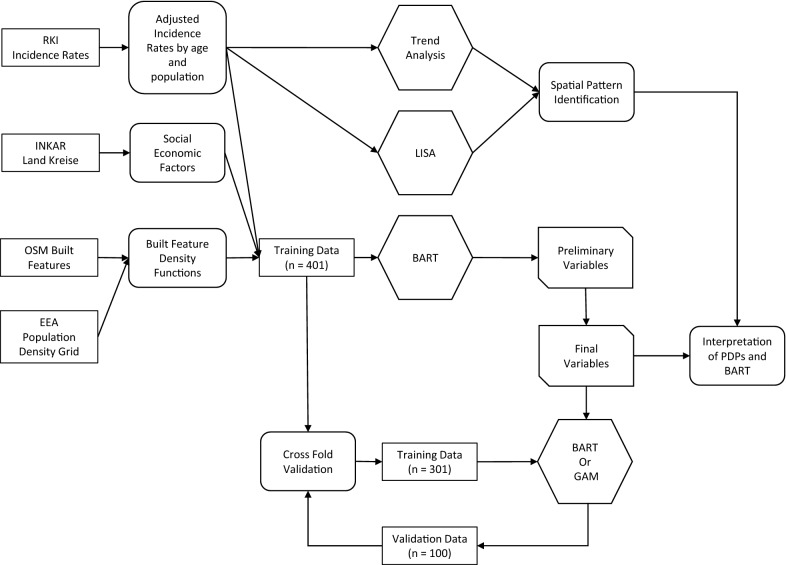
Methodology to examine patterns of COVID-19 incidence as defined by spatial, socioeconomic, and built environment features and characteristics. RKI: Robert Koch Institut; INKAR: Indikatoren und Karten zur Raum- und Stadtentwicklung [Indicators and maps for land and urban development]; OSM: OpenStreetMap; EEA: European Environment Agency; BART: Bayesian Additive Regression Trees; GAM: Generalised Additive Models; PDP: Partial Dependence Plot

We followed a linear methodology, as shown in Fig. 2, comprising data acquisition and preprocessing, spatial modelling, and aspatial modelling. County-level COVID-19 incidence data published by the Robert-Koch-Institute were downloaded through the publicly-accessible NPGEO-DE platform [37]. Socioeconomic data for Germany were collected through the INKAR (Indikatoren und Karten zur Raum- und Stadtentwicklung) data portal [38]. Built environment features were downloaded from OpenStreetMap [39] and the German Bundesamt für Kartographie und Geodäsie [40] data. Population densities were derived from the European Environment Agency’s 100-metre resolution Population Density Grid. Exploratory analysis of the geographic patterns was then undertaken using a geographical trend analysis and Local Indicators of Spatial Association (LISA). Finally, variable selection was conducted using Bayesian Additive Regression Trees (BART), where the most influential spatial, social-economic, built environment variables were selected for further interpretation in the context of the COVID-19 epidemic in Germany as of 1 April 2020. A 40-fold cross validation was conducted on the final BART outputs to assess prediction accuracy and model fit.

### Data acquisition and preprocessing

#### Incidence rates

COVID-19 incidence were downloaded on 1 April 2020, comprising a table of confirmed cases (N = 57,298) by county (N = 401) from the first case on 28 January until 31 March, comprising patient age group, sex, county of primary residence (NUTS-3), and the date at which the confirmed case was reported to the local health authority. Neither the date, location, nor means of infection were recorded.

Due to high spatial variation of age distributions in Germany, this analysis uses age-adjusted incidence rates. The age groupings used by the Robert-Koch-Institute for COVID-19 case reporting differ from those reported in population datasets; we therefore estimated age distributions for every county in the study area (N = 401). Based on the existing INKAR data, samples for each of the original age groups with sample sizes corresponding to the group’s proportion of the total county population were simulated. Those samples were then used to approximate an empirical cumulative distribution function for the entire age distribution, from which the probabilities for the new age groups congruent with those of the RKI were derived. These estimated probabilities were then multiplied by the municipality population to acquire an estimated absolute number of persons per age group. Our R code is available on GitHub [41]. The results were manually cross-checked against INKAR population data for validation and exhibited less than 2% error. With the resulting base population distributions we directly adjusted municipal incidence rates to the German standard population and natural-log-transformed the result to improve the distribution of rates for statistical analysis. The resulting rates were mapped for visualisation and spatial analysis.

#### Socioeconomic data

The socioeconomic datasets were acquired using the INKAR data access tool, which comprises social, demographic, and economic characteristics of counties collected by various ministries, the federal states, and the municipal governments, and is validated and managed by the German federal government. The dataset includes a diverse set of indicators in the fields of economics, demography, education, and other social data.

#### Built environment densities

OpenStreetMap data for Germany for each selected built environment feature type were downloaded in April 2020 as separate vector files from Geofabrik [39] and were used as the primary dataset for constructing our built environment variables. For modelling purposes, we separately computed a peak density value for each feature type in each county (e.g., airports, train stations, grocery stores, parks). To calculate the peak densities, we constructed a novel spatial density function to account for each feature type’s unique spatial structure, based on an heuristic approximation of geographical accessibility for each county population. This algorithm accounts for both the number and relative proximity of features of each type in each county [42], which were calculated using the Kernel Density Estimates function in the R package spatstat [43]. We created a custom parameterisation for each built environment feature within each county, calculated as the optimal bandwidth $h_{opt}$:

$$\begin{aligned} h_{opt} = \bigl [\frac{2}{3n}\bigr ]^{1/4}\sigma \end{aligned}$$

where $\sigma$ is the standard distance of all features within a given county and n is the total count of the selected feature type within that county [44]. A logit link function was then applied to estimate the optimal bandwidth for each county, selected in order to reduce biased weighting of spatially dense clusters of features at the expense of smaller clusters, e.g., in small towns and villages where person-to-person transmission is also likely to occur.

The calculated densities were then summarised for each feature type across each county, and each respective maximum density value was extracted for statistical modelling, based on the assumption that maximum densities provide a better approximation of person-to-person transmission than means or medians (e.g., in mostly rural counties with a small, yet very dense town, as is common in many regions of the study area).

### Exploratory spatial modelling

Local indicators of spatial association (LISA) was used to assess whether there was spatial clustering of log adjusted incidence rates for Germany. LISA is an exploratory tool used to statistically assess geographical clustering of high and low values in a dataset [45]. LISA calculates local spatial autocorrelation at each individual county using a single variable, enabling the quantitative estimation of local spatial clustering [45], essentially indicating how similar an observation is to all other observations within a defined radius [46]. We used LISA to identify statistically significant hot spots (clustering of high values), cold spots (clustering of low values), and spatial outliers (e.g., a county with high rates that is within a low-rate cluster). LISA was calculated using ArcGIS 10.7.1 [47]. The distance band (radius of the spatial weight function) was determined by calculating the average distance between all county centroids and an inverse distance squared parameter was used to define the spatial weighting function, selected to ensure higher weights were given to nearer counties.

Exploratory spatial trend analysis of adjusted incidence rates was conducted to identify spatial structure in the data. Trend analysis is the identification and description of a univariate spatial pattern using multiple regression, where the response variable is the variable of interest (adjusted incidence rate) and the predictor variables are longitude and latitude [29, 48, 49]. The results can be interpreted as a global indicator of the spatiality of response variable [50].

### BART

We elected to use a Bayesian modelling approach, which has the advantage (among others) of not being bound to the assumption of parametric parameter distributions, while facilitating model parameterisation based on prior data and/or iterative selective sampling of observed data distributions [51]. This approach allows for a reduction of bias and variance and for minimizing error when analysing small samples for inferential and prediction/classification problems [34, 52].

In order to identify important socioeconomic and built environment covariates with COVID-19 incidence rate, a Bayesian Additive Regression Trees (BART) model was selected. BART is a machine learning tool that iteratively creates regression trees with variable hyperparameter distributions (e.g., number of nodes, tree depth) [53]. The parameter distributions are recorded from multiple iterations using a Metropolitan-Hastings sampling algorithm, as all parameters and hyperparameters are not assumed to be parametric [53]. Unlike most ensemble methods, BART computes Bayesian posterior distributions to approximate the nonparametric model parameters and selects a strict error variance parameter to reduce the risk of overfitting. Additionally, BART has been shown to be effective at finding structure in high dimensional data [54] lending itself to be an exploratory method. further insights with the addition of an internal variable reduction method to emphasise important variables [53]. We used further measures to prevent overfitting and to select the optimal independent variables and hyperparameters by running iterative k-fold cross-validations with 5 to 20 folds. The BART Machine models were run in RStudio (v.1.2) using R (v.3.6.3) [55] with the BARTmachine package [53].

For model specification, we entered the natural-log-transformed age-adjusted incidence rates as the response (dependent) variable and all socioeconomic and built environment variables as candidate explanatory (independent) variables. Explanatory variable inclusion was determined through iterative cross-validations, in which each successive permutation of a BART model was assessed according to its error variance and RMSE to derive the model with the highest prediction performance. Overfitting is penalised with the BART model from its prior on error variance which limits the weights given to trees with small ${\sigma ^2}$ values [53].

Variable importance plots were generated from the BART model, which displays a quantitative metric of a variable’s relative influence on model predictions, compared to all other variables [53]. We also generated Partial Dependence Plots (PDPs), which are graphical outputs that illustrate the marginal effect of each independent variable on the response variable [56–58]. A PDP only displays the marginal effect of each independent variable in relation to the influence of all other independent variables, and should be interpreted as exploratory [53].

To assess how the final COVID-19 BART model should generalize to an independent data set, out-of-sample cross-validation was conducted on the 31 Final Variables that our BART model predicted The original training data were randomly split into training (n = 301) and testing (n = 100) subsets and a new BART model with 31 variables was computed. The model of the training subset was then used to predict the out of sample values of the testing subset. Finally, the actual values and the predicted values were compared with a linear regression analysis and the resulting RMSE and ${R^2}$ were calculated. Model outputs were validated using the test data and the resulting RMSE was calculated. This step was iterated 40 times, and an average RMSE was computed for all 40 runs to internally validate our predictions [56, 59].

### GAM

Due to nonlinear relationships expressed by model covariates, General Additive Models (GAMs) provide a useful semiparametric technique for modelling nonlinear associations [60]. GAMs operate as an extension of GLMs, but allow for the inclusion of smoothing terms, which can be explained by the following general form [61]:

$$\begin{aligned} \quad g (\mu _{i}) = A_{i}\gamma + \Sigma _{j}f_{j}(x_{ji}), y_{i}\sim EF(\mu _{i},\phi ) \end{aligned}$$

where $A_{i}$ is the ${i^{th}}$ row of the parametric model matrix of the model with parameters $\gamma$, and the smooth terms $\Sigma _{j}f_{j}(x_{ji})$ constitute the nonparametric part of the model. The response variable $y_{i}$ with the expected value $\mu _{i}$ follows a distribution from the exponential family, for which a link function $g (\mu _{i})$ can be specified [61]. The GAM model predictor variables were the top ten variables that were determined from the BART model’s variable importance plots, and the natural log-transformed age-adjusted incidence rate was selected as the response variable. Since the transformed incidence rates are approximately normally distributed, a gaussian model with an identity link function was used. The applied the GAM equation can be described as:

$$\begin{aligned} \log (AdjRate)_{i} = \beta _{0} + \Sigma _{j}\beta _{j}x_{ji} + \Sigma _{j}f_{j}(x_{ji}) + f(x_{1i},x_{2i}) \end{aligned}$$

where $\log (AdjRate)$ is the expected value of the natural log-transformed age-adjusted incidence rate, and the intercept is given by $\beta _{0}$. $\Sigma _{j}\beta _{j}x_{ji}$ accounts for the parametric model part to assess linear effects. For the nonlinear predictors $\Sigma _{j}f_{j}(x_{ji})$ thin plate splines were used as basis functions. For the county centroid coordinates a bivariate, isotropic smoothing term $f(x_{1i},x_{2i})$ was used, containing latitude and longitude as variables $x_{1}$ and $x_{2}$ respectively. A second GAM model was conducted without the latitude and longitude variables to reduce the concurvity amongst the socioeconomic and built environment variables.

## Results

**Fig. 3:**
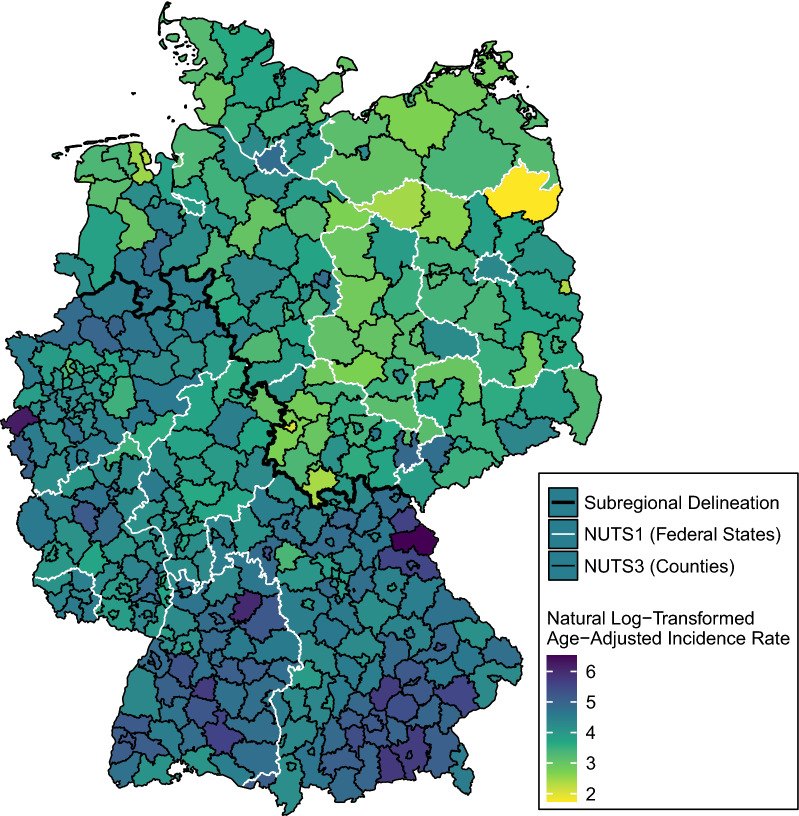
Natural log-transformed age-adjusted incidence rates of COVID-19 as of April 1st

There are 401 counties in Germany; as shown in Fig. 3, these vary in size, such that the counties in Southern Germany are generally smaller with higher population densities. Natural log-transformed age-adjusted incidence rates are shown, indicating spatial variation between the northeast and south-southwest of the study area.

### Spatial trend and LISA

**Fig. 4:**
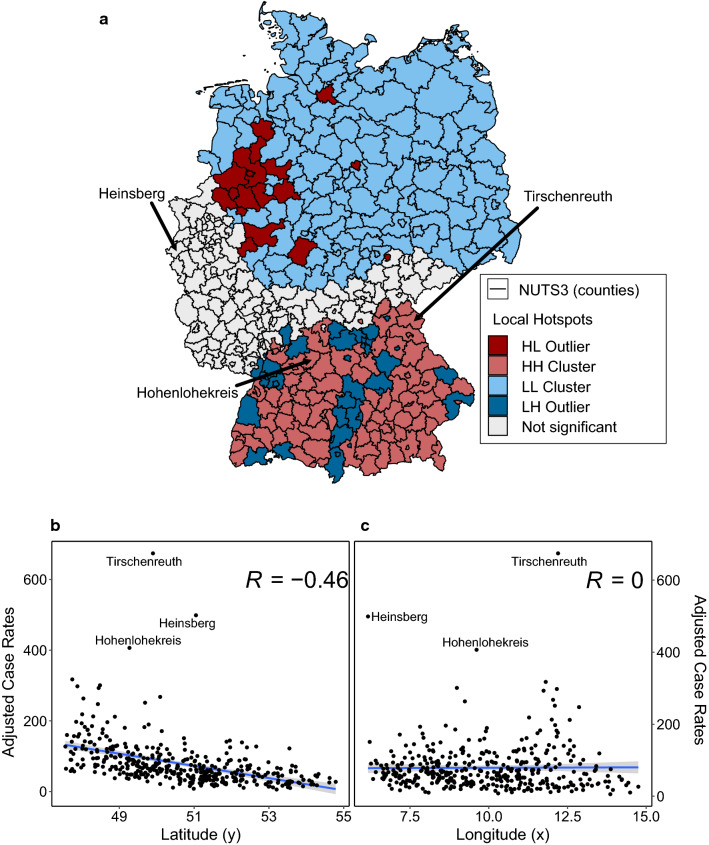
Trend analysis of age-adjusted incidence rates of COVID-19: adjusted incidence rates map **a** displays the LISA results, indicating significant spatial clustering in the study area. High-High (HH) indicates clusters of high rates, Low-Low (LL) indicates clusters of low rates. High-Low (HL) values represent individual counties with a high rate, but are surrounded by counties with a low rate, and Low-High (LH) is its inverse. The scatter plot with Pearson’s Correlation Coefficient indicates an association with **b** latitude, but not **c** longitude

The results of the trend analysis (Fig. 4b, c) indicate no apparent correlation between longitude and incidence rates, as can also be observed in the map (Fig. 4a). However, latitude does exhibit a weak-to-moderate correlation (*R* = −0.46), such that rates (shown as vertical extrusions on the map) indicate higher rates in the south. The LISA results (choropleth map in Fig. 4a) indicate a large cluster of high rates was observed in the south, whereas the northern and eastern regions exhibit a cluster of low rates. These constitute two major clusters with several outliers, for example, some counties (e.g., Erlangen-Höchstadt and Oberallgäu) are low-rate outliers. An east-west corridor with no significant spatial clustering is observed, dividing the north-eastern and southern clusters.

These trend analysis and LISA results indicate the presence of two distinct spatial patterns within Germany, enabling the classification of all federal states into two regions for the subsequent analysis: High-Rate Regions (HRR, referring to the southern cluster) and Low-Rate Regions (LRR, referring to the northern cluster). These regions are separated by a thick black line in Fig. 3.

### Regional comparison

The North/LRR accounts for 48.5% (173,287 $\hbox {km}^2$) of the total land area and 35.6% of the population, and the South/HRR for 51.5% (183,887 $\hbox {km}^2$) of the total land area and 74.4% of the total population of Germany.

**Fig. 5:**
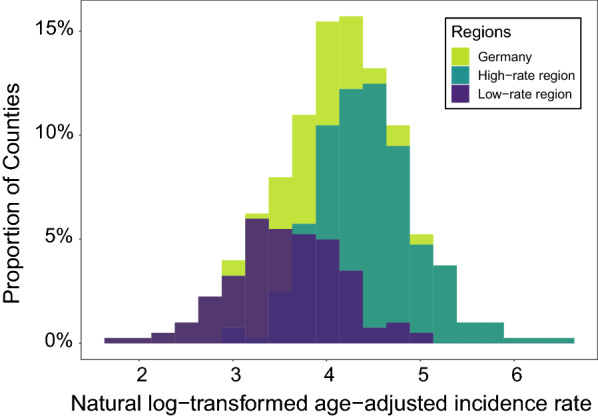
Histogram of the high rates region and low rates region subsets of COVID-19 incidence rates

The adjusted incidence rates exhibit two distinct distributions when regionally classified by LRR and HRR (Fig. 5), indicating that LRR and HRR are two distinct patterns. For ease of interpretation, further examination of the two regions is described using untransformed, age-adjusted values (Table 1).

The south western region has a greater representation of higher incidence rates where $\overline{\mathrm{X}}$ = 98.96 cases per 100,000 and $\sigma$ = 70.73 and minimum and maximum incidence rates of 20.60 and 673.93. The northern region has less proportion of counties, with the $\overline{\mathrm{X}}$ = 41.92 and $\sigma$ = 25.95 with county-level rates ranging from 5.76 to 139.10. LRR Germany’s max value of 139.10 (Mühldorf a. Inn), was lower than 42 counties in HRR, where the max was 673.93 (Tirschenreuth).

**Table 1:** Descriptive statistics for untransformed age-adjusted incidence rates per 100,000 for Germany and for the low rate and high rate subregions, and the differences between subregions

Adjusted rates region	N	Mean	Median	SD	Range	Min, Max
Germany	401	79.04	64.07	65.01	668.17	5.76, 673.93
high-rate region	261	98.96	81.17	70.73	653.33	20.60, 673.93
low-rate region	140	41.92	35.07	25.95	133.34	5.76, 139.10
Difference	$\mid$121$\mid$	$\mid$57$\mid$	$\mid$46.1$\mid$	$\mid$44.8$\mid$	–	–

### BART results and validation

The initial BART model included 366 independent variables (longitude, latitude, federal state (Bundesland) and NUTS2 region, and all socioeconomic and built environment variables). The response variable was the age-adjusted incidence rate per 100,000 residents.

**Table 2:** BART model summary statistics with internal validation

Model name	Number of variables	RMSE	Pseudo-Rsq	Shapiro-wilk test of normality of residuals (p-value)
Preliminary variables	366	0.23	0.8862	4e−05
Final variables	31	0.36	0.7341	0.00012

Comparisons were made between the preliminary variables (n = 366) and the final variables (n = 31)

Two BART models (Table 2) were produced to predict COVID-19 incidence rates. The preliminary model (366 variables) produced a root mean square error (RMSE) of 0.23 log-transformed age-adjusted incidence rate per 100,000 with a range of 2 to 6 and a pseudo $R^2$ of 0.886. This cross-validated model accounts for 88.6% of the variability in incidence rates, indicating a robust prediction.

To decide on the subset of variables that are contributing to the largest proportion of model influences, the variable selection function in the BART package was implemented [53]. Of the 366 variables, this variable reduction method removed all but 31 variables, as they were deemed the most important to the model’s predictions. This saw a reduction in pseudo $R^2$ from 0.886 to 0.734, equating to a 15% reduction in explained variability. The RMSE correspondingly increased to 0.36, indicating that the final model predicted age-adjusted incidence rates of COVID-19 for German counties with an accuracy of +/− 1.3 cases per 100,000. The residuals of both models were found to be normally distributed and exhibited no geographical clustering. The cross-validation was completed with 40 folds, and the resulting $R^2$ was 0.57 with an RMSE of 0.46, equating to a mean error of 1.58 cases per 100,000.

The density of Christian churches contributed the greatest number of tree splits in the final BART model. Latitude and Longitude respectively ranked second and third, indicating the importance of the spatiality in predicting incidence rates, as also observed in the trend analysis and LISA results. This spatial pattern is based on the x and y coordinates for the county centroids, which the BART model used to split decision trees for rate prediction. Socioeconomic variables account thereafter for a considerable proportion of the variability in rates, the strongest of which was Voter Participation rate. The remaining socioeconomic and built environment variables are described in rank order in an Additional file 1 in the appendix.

### Partial dependence

**Fig. 6:**
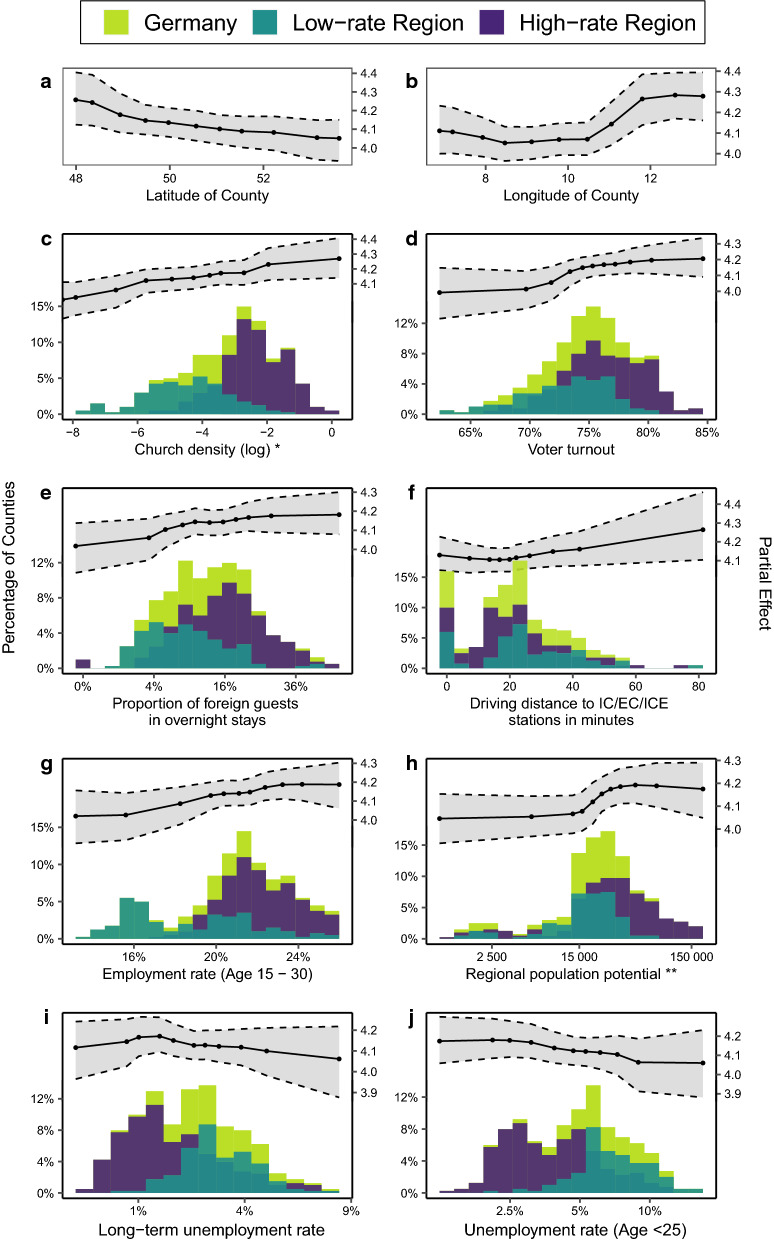
Partial Dependence Plots (PDP) of the 10 most prevalent variables in the final Bayesian Additive Regression Tree (BART) model. Histograms are shown for the entire country (green), for only the low rates region (LRR, teal), and for only the high rates region (HRR, purple). The PDPs indicate marginal changes in the predicted (log-transformed, age-adjusted) incidence rate per 100,000 residents (upper y-axis) for different values of each independent variable (x-axis)

The ten most important variables from the BART model were selected for further description. All variables and their summary statistics are listed in the Additional file 1: appendix. The partial dependence plots and region-specific histograms are shown in Fig. 6. We observed that increase in latitude (Fig. 6a) is associated with a strong marginal decrease in COVID-19 incidence rate, indicating that the model is accounting for the spatial pattern observed in the trend analysis. A partial dependence for longitude (Fig. 6b) indicated that farther east latitudes are associated with higher incidence rates. This trend is observed to be non-linear, rather quadratic. High rates along the Austrian border appear to account for this partial dependence.

LRR was observed to feature lower densities of Christian churches than HRR (Fig. 6c), and a higher density is associated with an increase in COVID-19 incidence rates. The voter participation rate (2017 national election) features minor differences between the two subregions (Fig. 6d) and the PDP indicates a positive relation between voter participation and incidence rates with a gradient increase between the 20th and 40th percentiles (73.5% and 74.3% participation). The histograms of the proportion of foreign guest overnight stays compared to the total number of overall stays (Fig. 6e) slight differences between the two subregions, accompanied by a positive association observed in the accompanying PDP. Conversely, there appear to be no significant differences in the distributions nor any significant observable partial dependence for long-distance train stations (Fig. 6f).

The regional population potential (Fig. 6h) measures the likelihood of direct interactions to occur between inhabitants [38]. The PDP indicates small marginal changes in incidence rates for low values of regional population potential, which can be interpreted as evidence that in counties with a lower probability of human interaction, there is a lower probability of viral contagion. The greatest increase in partial dependence is observed between the 20th and 80 percentiles of regional population potential index scores (14,016 to 47,067), indicating a strong non-linear effect of this variable on incidence rates. Both long-term unemployment rate and unemployment rate ages 15 to 30 exhibit differences between the study subregions, and both indicate minor partial dependence, such that higher unemployment rates correspond with lower observed COVID-19 incidence rates.

### GAM results and validation

Initially two base models were fitted, one with the ten variables that attained the highest variable importance in the BART model, and one with eight variables, for which the variables for longitude and latitude were excluded. In both models the residuals showed no association with the response variable. The model including latitude and longitude showed high concurvity values and suffered from lower significance for the non-spatial variables (except church density). Further modelling was conducted on the eight non-spatial variables and the final GAM model was chosen by selecting the model with the lowest RMSE (as validated by a 1000 fold-cross validation) and AIC scores. Among the final model candidates, the non-spatial base model and the model including employment rate of persons ages 15 to 30 and unemployment rate under 25 as single terms display the lowest AIC scores, the lowest RMSE value of 0.485 and an $R^2$ of 0.557 with the minimum value varying between the two test runs. This model reduced concurvity and model complexity, while performing equally well across all criteria examined here, it was chosen as the final model.

## Discussion

### Intra-population contagion

The level of response to COVID-19 has been adapted to the current outbreak with increasing severity, with several initial steps taken in May 2020 to reduce restrictions [62]. Local measures have included encouraging and or mandating a minimum interpersonal distance 1.5 m [11], closing schools, colleges, universities, community centres, and daycare centres, and a widespread implementation of “work-from-home” arrangements. These policies have almost certainly reduced the potential spread of SARS-CoV-2 in the study area, although this study focuses on a snapshot of data from 1 April 2020. The results presented herein may be valuable not only for improving our current understanding of transmission dynamics and population vulnerability, but also for informing outbreak control measures and targeting high-risk areas.

The transmission of COVID-19 is facilitated through interactions occurring at multiple scales as they interact with vertical and lateral transmission. The scale of interaction is defined by its own set of distinct spatial patterns [63]. By examining the assemblage of each pattern, researchers can eventually define the structure of an otherwise prohibitively complex process [64, 65]. In this case, the underlying process of interest is the vertical transmission of intra-population contagion in Germany at population and sub-population scales. The population’s members in this instance can be defined by social, cultural, economic, and spatial factors [66, 67] as expressed by the county units.

The LRR and HRR groups defined in this study exhibited very distinct and contrasting characteristics that were observed to influence the higher observed rates in the South-West and the lower rates in the North-East. This regional distinction and the variable selection generated using BART enabled us to achieve high model accuracy and define a spatial pattern related to intra-population contagion as expressed by the sub-population observations.

The most important variables identified through our methodology merit further discussion. Higher densities of churches were observed in the HRR, which were identified as being the most important environmental variable for predicting COVID-19 incidence rates. However, this does not necessarily indicate that the churches themselves are the loci of transmission, rather, we suggest that this feature of the built environment indicates locales with higher walkability where more interpersonal interactions may take place, for example, due to higher social connectivity and community engagement, particularly among senior and elderly populations, who comprise the majority of Christian church attendees in our study area and are more likely to be diagnosed with COVID-19.

Similarly, features of transportation networks such as long-distance train stations may serve predominantly as an indicator of a community’s connectedness (inter-population invasion), as well as serving as nodes where high densities of travelling persons increase the probability intra-population contagion [21].

### SES and built environment

The transmission of COVID-19 can occur through both direct and indirect interpersonal contact [68, 69]. The frequency and proximity of interactions between individuals is therefore a primary determinant of infection risk. The nature and configuration of the social and built environments therefore are likely to be significant covariates of infection risk, and consequently, the resulting geographical distribution of incidence.

An key driving assumption in this study is that higher built environment densities will correspond with increased direct and indirect contact between persons, and decreased proximities [70]. However, our analysis revealed only one built environment variable that contributed to an heuristically significant proportion of the variability explained in our models: the density of Christian churches. It is therefore crucial to underscore the generalised nature of how built environments are assessed in contemporary methodologies, specifically, that individual features do not necessarily constitute precise loci of transmission, rather, that they may serve as proxies for understanding the configuration of the built environment and difficult-to-measure characteristics of local populations (e.g., community connectivity amongst elderly populations).

Similarly, the socioeconomic variables highlighted in section 3.4 and listed in Additional file 1 appendix may serve to characterise local inter- and intra-connectedness, in addition to describing measurable characteristics of a population (e.g., age distributions). In counties where the incidence rate of COVID-19 is high we postulate that those variables proxying social interactions also exhibit high values, because they increase the potential of spreading the virus through local instances of viral transmission.

Interestingly, three variables related to labour market structure emerged as highly important in predicting COVID-19 incidence rates: unemployment rate, unemployment rate among persons ages 25 or younger, and the employment rate of persons ages 15 to 30. The spatial distributions of these variables also reflect the geographical distribution of labour market participation across Germany, and our models and the resulting partial dependence plots indicate a negative correlation between employment rates and COVID-19 incidence rates. This may be explained by the mechanics of social exclusion and stratification, such that employed persons are more likely to have a more differentiated social network than unemployed persons [71]. However, social exclusion and relative isolation caused by unemployment may lead to a more closely knit socio-spatial milieu [72–74]. Accordingly, we would expect that higher employment rates and lower unemployment rates are both correlated with a higher number of social interactions and reduced interpersonal proximities, consequently amplifying the potential spread of SARS-CoV-2. Very recent research is poised to illuminate how and when the actors engaged in social service work are addressing changes in the social settings and consequent vulnerability experienced by socially excluded members of society [75, 76].

Spatial interconnection is represented in our final model primarily by access to long-distance train stations, the proportion of foreign guests, and the regional population potential variables. We therefore hypothesised that the socio-spatial variables would be important in the resulting BART models. The partial dependence plots for these variables also correspond to our heuristic expectations, for example, that voter participation and access to intercity train stations would exhibit positive partial dependence. However, these variables did not exhibit differences in their distributions between the two study regions, except for the proportion of foreign guests, which provides weak correspondence to a differentiation between the regions.

### Modelling

The concept of parsimony is central to new modelling studies, particularly within an exploratory framework [77, 78]. However, when examining large, multidimensional datasets in an exploratory fashion more complex methods are necessary, in order to detect potential patterns and associations [79]. The observations that are made through simpler, often parametric models are critical in interpreting and contextualising results from modern exploratory data-mining models, which are often obscured behind the black box of machine learning [32, 34]. In this context, the robustness thesis can therefore be considered a companion to parsimony, in that is asserts that a method is robust if observations made with a simpler model are also present in a different or more complex model [32].

This study demonstrates a novel methodology for systematically exploring geospatial patterns of EIDS while building ideas of the robustness thesis into our procedure/methodology. Early exploratory analysis (as seen with the trend analysis) enabled us to gain confidence in the subsequent, more complex model’s explanation of the spatial pattern [32]. These early exploratory tests can also be used to validate assumptions about the spatial nature of a dataset while providing a method for separately validating trends observed in machine learning results. Latitude and longitude represent simple spatial variables that can help define global functions of an observed spatial pattern of an epidemic, and enable researchers to parameterise models accordingly. For use of this study, we assumed there were no causal effects that are associated with the X and Y variables, instead, these variables were used to validate assumptions we witnessed in our trend model. This approach emphasises necessity for critically interrogating data and methods in order to be confident in our model outputs. As we try to ensure that our data heuristically correspond with the process or target under examination [27, 34, 80], we provide space for hypotheses to be generated that question these intricate data and process relations.

The BART modelling demonstrated that although many variables can be used as inputs, the majority of variability explained will largely be determined from a subset of all variables, whenceforth only a marginal decrease in accuracy will be observed [58]. The preliminary model decreased from 366 to only 31 variables in the final model, and the $R^2$ only exhibited a proportionally small decrease from 0.89 to 0.73, with very minimal differences in variable importance among the top 10 variables shown herein. Because the removal of 335 variables contributed to a 0.16 reduction in $R^2$, a more thorough investigation can be conducted on the remaining 31 variables. The cross validation results indicate that even when we further subsetted the data (n = 301 and n = 100), the resulting $R^2$ values remained relatively high ($R^2$ = 0.57) with an RMSE of 1.58 cases per 100,000.

The inclusion of the GAM models allowed for a comparison for the efficiency and accuracy of the BART model. As an exploratory tool, GAM was overburdened by the complexity of the data and the amount of variables. The BART analysis was not only required to determine variables for modelling, but the GAM model would often express too much concurvity when purely spatial variables such as latitude and longitude were used. The inclusion of the latitude and longitude were key indicators to express the patterns described by the trend analysis. However, once exploration is conducted, we suggest that future studies use the GAM modelling to further understand the associations that have been presented by the BART modelling.

Another important feature of this study is the use of partial dependence plots to assess marginal effects on the response variable for different values of an independent variable. For example, a visual examination of the PDPs uncovered patterns that were not evident from the maps and trend analysis. The use of PDPs for spatial-epidemiological analysis is therefore recommended as a means of adding a layer of interpretability to machine learning models.

### Study limitations

The modelling approaches selected for this study feature several key limitations that may have impacted our results. These limitations are explored in more detail in the methodology papers referenced herein, but several merit mention.

The use of administrative boundaries still requires that our results be considered in light of limitations such as the Modifiable Areal Unit Problem (MAUP) [18]. For example, it is unclear whether there are significant differences in COVID-19 rates and population characteristics between high-incidence counties on either side of Germany’s borders with France, Switzerland, and Austria. The next phase of this project intends to expand this methodology to include cross-border effects, using NUTS3 data from multiple countries in continental Europe. In addition, this study is unable to determine whether the origin of each new COVID case is locally or internationally acquired. We have discussed variables that can be used as indicators for global (proportion of foreign guest stays) or local (unemployment rates), however origin is still unknown.

A significant challenge in the modelling of many EIDs is that the true population incidence and prevalence are unknown, largely due to asymptomatic individuals, different testing rates and protocols, misdiagnosis, and differences in reporting protocols. This limitation may provide additional challenges when seeking to conduct analyses that include multiple countries, and must be taken into consideration during comparative or multi-site studies.

Although BART provides a useful non-parametric means of exploring potential associations in large, multidimensional datasets, the use of Markov Chain Monte Carlo to generate prior distributions for all parameters and hyperparameters requires a strong penalty against overfitting; it is unclear whether the built-in penalty against sigma-squared is sufficient. This study used an internal cross-validation approach to account for overfitting, however, an independent validation dataset could be used in future studies to assess these effects. Additionally, because the Metropolitan-Hastings algorithm uses a random seed, some variation in model repetitions is observed and exact replication of results requires additional parameterisation. In order to address this limitation, we provide pre-set seeds in our code, linked in this article. The use of regression trees with many nodes also increases the probability of spurious splits occurring, although BART has the advantage of using the sums of multiple iterations to reduce these effects. However, these instabilities require that BART be used as an exploratory tool, and not in a confirmatory manner. For this reason, the use of GAMs or other robust regression techniques is vital for assessing and confirming BART results.

Although exploratory results determined that no other patterns existed on other administrative scales (NUTS1 and NUTS2), this study focussed primarily on the NUTS3 (county) level of geography, limiting our model interpretations and ability to generalise from the data. Additionally, it has been shown that the spatial scale of data analysed dictates the spatial granularity of a study, which could in turn limit the ability to identify the correct scale for the process under investigation [18].

## Conclusions

This study provides a first step towards understanding the spatial, socioeconomic, and built-environment structure of COVID-19 incidence across Germany. Through the BART modeling and variable importance, 10 variables were identified as being very important for explaining variance in incidence rates: church density, latitude, longitude, voter participation, foreign guests, accessibility by intercity rail, employment rate for ages 15–30, population potential, long term unemployment rate, and unemployment under age 25. When split spatially into northeastern (LRR) and southwestern (HRR) regions, clear trends and patterns emerged that assisted with interpreting the most important independent variables and their respective influence on the prediction of COVID-19 incidence rates.

Additionally, this study provides an example of the utility of partial dependence plots for gaining more detailed insights from machine learning models. Especially when combined with other spatial tools, integrating these approaches holds strong potential for elucidating a more complete explanation of epidemiological patterns with greater precision and accuracy. However, a broader movement is required to establish process-based methods for disease and pandemic mapping [27] in order to ultimately improve outbreak prevention and control measures.

We encourage future machine learning studies to follow a similar level of data exploration as shown herein. This procedure facilitated a better understanding of how the produced model interpreted the input data by enabling the observation of spatial patterns in three increasingly complex representations (trend analysis to LISA to BART). This satisfied assumptions defined by the robustness thesis [32], while the splitting of the study area into geospatially relevant regions allowed for increased interpretability of machine learning model results and the partial dependence plots.

## Supplementary information

**Additional file 1.**

## Data Availability

INKAR data are available at https://www.inkar.de (gathered and aggregated from the BBSR and the ongoing spatial monitoring of the federal german institutions https://www.bbsr.bund.de) Built environment data are available at https://download.geofabrik.de/ COVID-19 case data are published daily by the Robert-Koch-Institut, and are available at https://npgeo-corona-npgeo-de.hub.arcgis.com/ R code available at https://github.com/CHEST-Lab/BART_Covid-19
