# Supplementary material for: A multimethod approach for county-scale geospatial analysis of emerging infectious diseases: a cross-sectional case study of COVID-19 incidence in Germany
Source: Int J Health Geogr. 2020 Aug 13;19:32. doi: 10.1186/s12942-020-00225-1 (PMC7424139; doi:10.1186/s12942-020-00225-1)
Supplement: Supplementary file 1 — Additional file 1. [file 12942_2020_225_MOESM1_ESM.docx]

# Additional file

Christopher Scarpone, Sebastian T. Brinkmann, Tim Große, Daniel Sonnenwald, Martin Fuchs and Blake Byron Walker


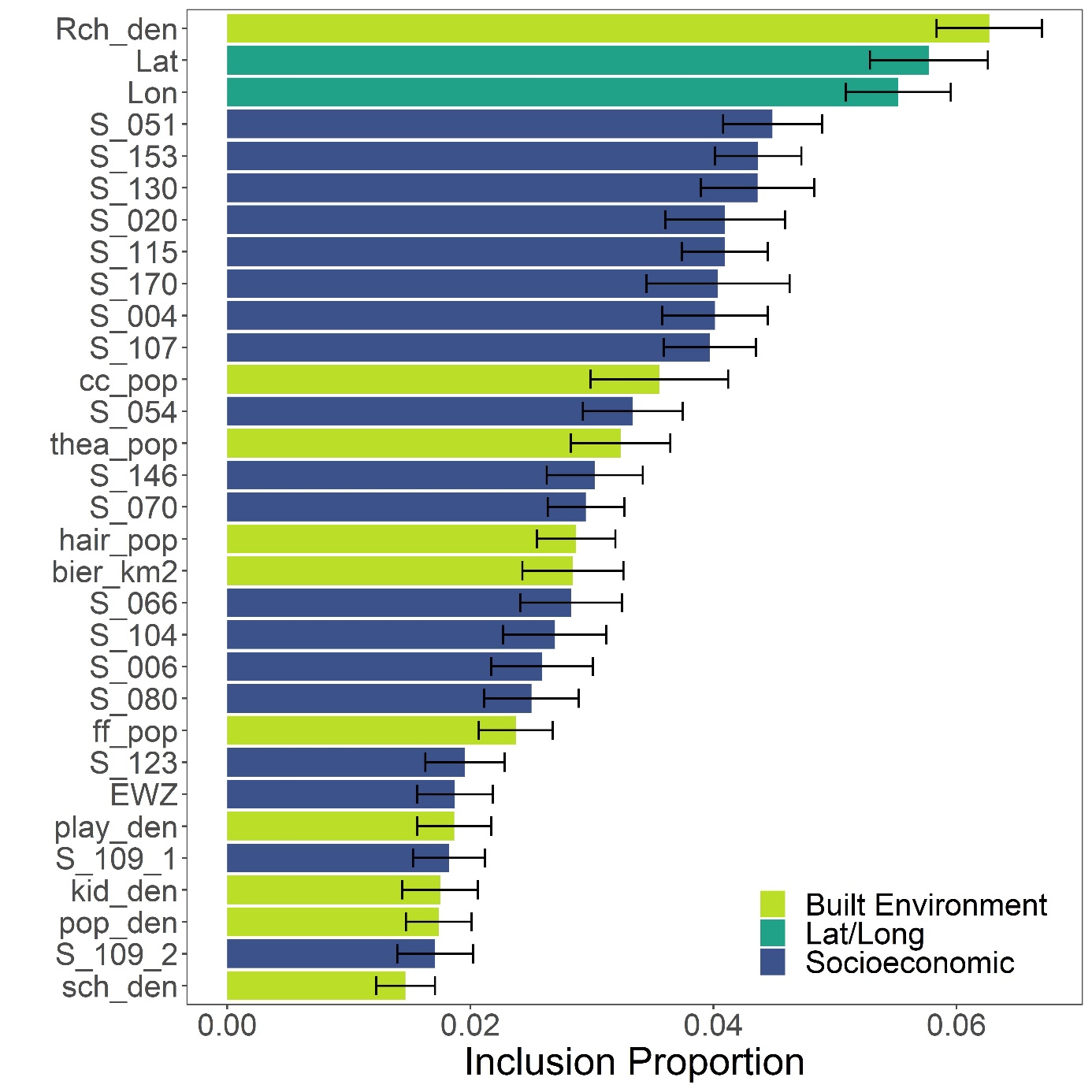


**Figure S1:** Variable importance plot, ranking model variables by the proportion of all decision tree splits (inclusion proportion) that were influenced by each variable in the final BART model. Each variable is classified as geographical (Lat/Long), built environment, or socioeconomic.

**Table S1:** Variable Explanation and Descriptive Statistics, ranking model variables by their importance.

| **Variable Code** | **Independent Variable** | **Variable Importance-Splits (95% credible interval)** | **Variable Importance-Trees (95% credible interval)** |
| --- | --- | --- | --- |
| Rch_den | Church density | 0.063 (0.058-0.067) | 0.047 (0.044-0.051) |
| Lat | Latitude | 0.058 (0.053-0.062) | 0.047 (0.044-0.049) |
| Lon | Longitude | 0.054 (0.050-0.059) | 0.046 (0.043-0.049) |
| S_051 | Voter participation | 0.046 (0.041-0.050) | 0.043 (0.040-0.045) |
| S_153 | Foreign guests in tourist establishments | 0.044 (0.040-0.048) | 0.042 (0.039-0.045) |
| S_130 | Reachability of IC/EC/ICE stations | 0.042 (0.038-0.045) | 0.042 (0.039-0.044) |
| S_020 | Employment rate 15-<30 | 0.041 (0.037-0.044) | 0.042 (0.039-0.045) |
| S_115 | Population potential  within 100 km radius | 0.041 (0.037-0.045) | 0.039 (0.036-0.042) |
| S_170 | Long-term unemployment rate | 0.04 (0.034-0.047) | 0.039 (0.036-0.041) |
| S_004 | Unemployment rate under 25 | 0.04 (0.034-0.046) | 0.038 (0.035-0.041) |
| S_107 | Tax revenue | 0.04 (0.035-0.044) | 0.037 (0.033-0.04) |
| cc_pop | Community_centre per capita | 0.036 (0.031-0.040) | 0.034 (0.030-0.037) |
| S_054 | Apprenticeship places | 0.034 (0.029-0.040) | 0.034 (0.031-0.037) |
| thea_pop | Theatre per capita | 0.032 (0.028-0.035) | 0.033 (0.029-0.037) |
| S_146 | Commuters with commuting distance 150 km and more | 0.03 (0.026-0.033) | 0.032 (0.029-0.036) |
| S_070 | Debtor quota | 0.03 (0.026-0.033) | 0.032 (0.028-0.035) |
| hair_pop | Hairdresser per capita | 0.03 (0.025-0.034) | 0.032 (0.028-0.035) |
| bier_km2 | Biergarten per km² | 0.029 (0.025-0.034) | 0.031 (0.027-0.034) |
| S_066 | Household income per capita | 0.028 (0.024-0.031) | 0.031 (0.027-0.034) |
| S_104 | Income tax | 0.027 (0.023-0.032) | 0.029 (0.026-0.032) |
| S_006 | Unemployed under 25 per 1000 15-25 | 0.026 (0.022-0.030) | 0.028 (0.025-0.032) |
| S_080 | Recreation area per inhabitant | 0.025 (0.022-0.029) | 0.027 (0.024-0.031) |
| ff_pop | Fast food places per capita | 0.024 (0.019-0.028) | 0.026 (0.023-0.029) |
| S_123 | Child poverty | 0.020 (0.017-0.023) | 0.023 (0.02-0.027) |
| EWZ | Population | 0.019 (0.016-0.022) | 0.022 (0.019-0.026) |
| play_den | Playground density | 0.018 (0.015-0.021) | 0.022 (0.020-0.025) |
| S_109_1 | Rural area | 0.018 (0.014-0.022) | 0.022 (0.019-0.025) |
| kid_den | Kindergarten density | 0.018 (0.014-0.021) | 0.022 (0.019-0.024) |
| pop_den | Population density | 0.017 (0.014-0.020) | 0.021 (0.019-0.024) |
| S_109_2 | Urban area | 0.017 (0.014-0.020) | 0.020 (0.017-0.023) |
| sch_den | School density | 0.014 (0.012-0.017) | 0.018 (0.016-0.020) |

**Table S2:** Explanation and Descriptive Statistics of ten most important variables used in the Partial Dependence Plots.

| **Variable Code** | **Independent Variable** | **Mean** | **Median** | **SD** | **Skew** | **Range** |
| --- | --- | --- | --- | --- | --- | --- |
| AdjRate | Age adjusted case rate per 100,000 | 79.04 | 64.07 | 65.01 | 3.64 | 5.8-673.9 |
| Rch_den | Church density | 0.055 | 0.096 | 0.124 | 2.81 | 0-1 |
| Lat | Latitude | 50.62 | 50.56 | 1.74 | 0.25 | 47.5-54.8 |
| Lon | Longitude | 9.86 | 9.78 | 2.02 | -0.87 | 6.2-14.8 |
| S_051 | Voter participation | 75.08 | 75.3 | 3.79 | -0.83 | 63.1-84.1 |
| S_153 | Foreign guests in tourist establishments | 14.38 | 12.7 | 9.69 | 1 | 0-49.2 |
| S_130 | Reachability of IC/EC/ICE stations | 21.93 | 21 | 15.38 | 0.63 | 0-79 |
| S_020 | Employment rate 15-<30 | 20.86 | 21.3 | 2.79 | -0.52 | 13.8-25.9 |
| S_115 | Population potential within 100 km radius | 33,367 | 24,587 | 30,682 | 2.13 | 1,062-174,293 |
| S_170 | Long-term unemployment rate | 2.4 | 2.2 | 1.49 | 0.97 | 0.2-7.6 |
| S_004 | Unemployment rate under 25 | 5.26 | 4.9 | 2.71 | 1.022 | 1.4-16.5 |
